# An improved *Shorea robusta* genomic DNA extraction protocol with high PCR fidelity

**DOI:** 10.1093/biomethods/bpad039

**Published:** 2023-12-09

**Authors:** Garima Mishra, Rajendra K Meena, Rama Kant, Shailesh Pandey, Harish S Ginwal, Maneesh S Bhandari

**Affiliations:** Division of Genetics & Tree Improvement, ICFRE-Forest Research Institute, Dehradun 248 195, Uttarakhand, India; Division of Genetics & Tree Improvement, ICFRE-Forest Research Institute, Dehradun 248 195, Uttarakhand, India; Division of Genetics & Tree Improvement, ICFRE-Forest Research Institute, Dehradun 248 195, Uttarakhand, India; Forest Pathology Discipline, Division of Forest Protection, ICFRE-Forest Research Institute, Dehradun 248 006, Uttarakhand, India; Division of Genetics & Tree Improvement, ICFRE-Forest Research Institute, Dehradun 248 195, Uttarakhand, India; Division of Genetics & Tree Improvement, ICFRE-Forest Research Institute, Dehradun 248 195, Uttarakhand, India

**Keywords:** *Shorea robusta*, polyphenols and polysaccharides, DNA extraction protocol, qualitative and quantitative, SSR markers

## Abstract

*Shorea robusta* (Dipterocarpaceae), commonly known as Sal, is an economically and culturally important timber species, known to contain a wide spectrum of polyphenols, polysaccharides, and other secondary metabolites in the tissues, which can interfere with the extraction of high-quality genomic DNA. In order to screen simple sequence repeat (SSR) markers and carry out other DNA-based analyses for this species in our laboratory, a high-throughput DNA extraction methodology was needed. Hence, we have optimized a simple, rapid, safe, and reliable high-throughput protocol for DNA extraction suitable for both fresh and dry leaves. The standardized protocol delivered good DNA yield of ∼1500 µg from 1 g of leaf tissue, with purity indicated by a 260 nm/280 nm absorbance ratio ranging from 1.70 to 1.91, which validated the suitability of extracted DNA and revealed reduced levels of contaminants. Additionally, the protocol that we developed was found to be suitable for polymerase chain reaction (PCR) amplification using microsatellite markers. Genome-wide characterization with SSR markers has been established in *S. robusta*, which further validates the protocol and its usefulness in DNA-based studies across the genus and/or family.

## Introduction

The availability of high-quality genomic DNA is a crucial prerequisite for molecular genetic analysis of forest tree species. When plant tissues contain high levels of polysaccharides, secondary metabolites, or polyphenolics, it can be problematical to extract intact, high-molecular-weight DNA for polymerase chain reaction (PCR), genomic blot analysis, fingerprinting, and other DNA-based investigations [1]. Many DNA-based experiments require the extraction and availability of pure genomic DNA predominantly in forestry tree species [2]. Obtaining high DNA quality is therefore a crucial factor in genomic research and most amplification-based assays since co-purified inhibitors can interfere with DNA amplification and limit the effectiveness of subsequent PCR analysis [3, 4]. One purpose of the DNA extraction procedure is, therefore, to reduce the amount of polyphenols and polysaccharide content [5]. On a commercial level, available DNA extraction kits have a greater throughput but are typically limited to particular classes of starting material. Furthermore, their availability and high pricing may be restricting considerations.

Among Asian dipterocarps, predominantly in the Indian sub-continent, *Shorea robusta* (Vern: Sal) is an ecologically sensitive and commercially valuable woody species whose genetic diversity and phylogeny are currently uncertain due to a scarcity of data [6]. It is a prominent high-quality tree species for commercial timber production for revenue generation, as well as an outstanding supplier of construction material, tannin, gum, and oil, alongside being used as fodder and firewood [7]. Sal is known to have a significant amount of polyphenolic content [8]. The coexistence of such substances leads to substandard quality and quantity of extracted genomic DNA. According to some sources, this occurs because phenolic compounds oxidize and covalently attach to proteins and nucleic acids during the homogenization phase of DNA extraction, rendering the DNA ineffective for most research purposes [9–11]. For example, there are several standardized protocols for the isolation of plant DNA [12–14]. Even though these protocols consistently yielded high-quality DNA from samples of other species, as determined by PCR, the majority of *S. robusta* samples consistently failed to support PCR after these protocols. Furthermore, even samples that initially yielded good PCR outcomes for Sal, failed to do so after being stored for a few months. The purity and quantity of genomic DNA isolated using these protocols did not prove to be up to par for this tropical forest species. Consequently, we found it necessary to develop a robust DNA extraction protocol for *S. robusta* that could deliver a suitable DNA concentration with high purity for routine PCR amplification. Recently, we carried out *de novo* low-depth genome sequencing and identification of reliable microsatellite markers in *S. robusta* through genomic DNA extracted with the aid of this method [6]. In the present work, we have standardized a modified high-throughput DNA extraction protocol and incorporation of an optimal concentration of a PCR additive for *S. robusta* that can yield relatively high-quality DNA for microsatellite markers studies.

## Material and methods

### Solutions, reagents, and equipment

- 100 mM Tris base (Make: G-Biosciences).
- 20 mM Ethylenediamine tetra-acetic acid (EDTA) (Make: G-Biosciences).
- 1.52 M Sodium chloride (NaCl) (Make: Himedia).
- 8 mM Ascorbic acid (Make: Himedia).
- 4% Polyvinylpyrrolidone (PVP) (Make: Sigma-Aldrich).
- 4% Cetyltrimethylammonium bromide (CTAB) (Make: G-Biosciences).
- 0.2% β-mercaptoethanol (Make: Himedia).
- 5 M Sodium acetate solution (pH = 5.2).
- Chloroform (Make: Sisco Research Laboratories Pvt. Ltd–SRL).
- Isoamyl alcohol (Make: Rankem).
- 70% Ethanol and 96% Ethanol (v/v) (Make: Changshu Hongsheng Fine Chemical Co., Ltd).
- Isopropanol (−20°C) (Make: G-Biosciences).
- TE buffer (consisting of 10 mM Tris–HCl, 1 mM EDTA; pH = 8)
- Liquid nitrogen.
- Mortar and pestle.
- Water bath (Make: Narang Scientific Works Pvt. Ltd).
- Centrifuge (Make: Eppendorf).
- Thermo-block (Make: Labnet International Inc.).
- Gel Rocker (Make: Genei).
- 2 ml and 1.5 ml microcentrifuge Eppendorf tubes (Make: Axygen).
- Gel loading tips (standard and sheared) (Make: Axygen).

### Leaf tissue sample collection and preparation

Ten fresh indigenous accessions of *S. robusta* were collected in February2022 from natural populations belonging to different provenance(s) across the Kumaun regions of Uttarakhand, India (Supplementary Table S1). Samples were immediately dried up with silica gel and brought to the laboratory of Genetics and Tree Improvement Division, ICFRE-Forest Research Institute, Dehradun, and stored at –80°C until extraction.

### Optimized extraction and purification protocol

Initially, the protocols described by Doyle and Doyle [15] and Sahu *et al*. [16] were both used for Sal genomic DNA extraction. The results obtained were, however, substandard and of low quality when compared to the protocol described herein after making modifications to the latter [15, 16]. Therefore, the optimized protocol was developed by varying the concentrations of ascorbic acid, CTAB, and PVP in the freshly prepared extraction buffers (Table 1). We have used two buffer methods: buffer I was prepared without CTAB and buffer II was prepared with CTAB. The other contents are the same for buffers I and II, except for the absence of CTAB in buffer I. Both buffers were used at different steps of the protocol. Here, we report the final standardized protocol, including compositions and conditions that gave the best results:

**Table 1.** Composition of extraction buffers used in different protocols.

Optimized double buffer method		Protocol by Sahu *et al*. (2012)		CTAB method by Doyle and Doyle (1990)
Without CTAB (pH = 8)	With CTAB (pH = 8)	Without CTAB (pH = 8)	With CTAB (pH = 8)	
100 mM Tris Base	100 mM Tris Base	100 mM Tris Base	100 mM Tris Base	100 mM Tris Base
20 mM EDTA	20 mM EDTA	20 mM EDTA	20 mM EDTA	20 mM EDTA
1.52 M NaCl	1.4 M NaCl	1.4 M NaCl	1.4 M NaCl	1.4 M NaCl
8 mM Ascorbic acid	8 mM Ascorbic acid	5 mM Ascorbic acid	5 mM Ascorbic acid	5 mM Ascorbic acid
4% PVP	4% PVP	2% PVP	2% PVP	2% PVP
–	4% CTAB	–	4% CTAB	3% CTAB

- Initiating the DNA extraction procedure, 1 ml extraction buffer solution (pH = 8) without CTAB (buffer I) that contains 100 mM Tris–Base, 20 mM EDTA, 1.52 M NaCl, 8 Mm V Ascorbic acid, and 4% (w/v) PVP (after autoclave) with 3 µl of β-mercaptoethanol in 2 ml Eppendorf tubes, was cooled down for 20–30 min at 4°C.
*Note*: Two tubes for each sample are maintained (preps are duplicated), that is (a) and (b), so that if one tube is mishandled or spilled, another copy is safe and can be used as a substitute and also using duplicates reinforce confidence in good results if they are visible in all replicates.
- Fresh leaves (0.5 g for each sample) in liquid nitrogen were ground into fine powder with a pre-chilled mortar and pestle (–40°C) and then the dried powder was transferred to the tube containing 1 ml of pre-cooled extraction buffer (buffer I). This was then incubated at 4°C for 30 min while gently inverting it 2–3 times to ensure adequate mixing.
- The tube was then centrifuged at 4°C and at 7000 rpm for 5 min and the supernatant was discarded. About 1 ml of preheated (60°C) extraction buffer with CTAB, that is buffer II with 3 µl of β-mercaptoethanol was added to the remaining tissues in the tube. This was incubated in a water bath at 60°C for 30–60 min with gentle inversion and vortexing.
- Subsequently, the samples were cooled at room temperature for 10 min. Then, 500 µl of chloroform-isoamyl alcohol (24:1) was added to the mixture and incubated for 5 min. After that, the tubes were gently inverted for 20–25 min to form an emulsion.
- The emulsion was then centrifuged at room temperature (25°C), and at 13000 rpm for 15 min. The upper (aqueous) phase was pipetted out and carefully transferred to a 1.5 ml sterile Eppendorf tube.
*Note*: Cropped pipette tips should be used for transferring the resulting supernatant to avoid mechanical shearing damage to DNA.
- An equal volume of chilled isopropanol (at −20°C) was added to the supernatant, gently inverted, and then incubated overnight at −20°C for precipitation.
- The following day, the mixture was centrifuged at 13000 rpm for 15 min at 4°C to form a pellet, and the supernatant was discarded.
- Next, 998 µl of 96% alcohol and 2 µl of 5 M sodium acetate (CH_3_COONa) were added to the pellet. Furthermore, the mixture was kept over the gel rocker for 60 min, followed by centrifugation at 13000 rpm at 4°C for 5 min, and then the supernatant was again discarded.
- For washing the pellet, 500 µl of 70% alcohol was again added, and the mixture was centrifuged at 13000 rpm at 4°C for 5 min and the supernatant was removed.
- On thermo-block, the pellet was then vacuum dried at 37°C for 10–15 min until dry.
*Note*: Excessive drying should be avoided as it can make it challenging to resuspend the pellet.
- Finally, the pellet was re-suspended in 100 μl of autoclaved TE buffer (consisting of 10 mM Tris–HCl, 1 mM EDTA; pH = 8).
*Note*: Chelators present in TE can affect PCR and restriction digests. DNA in TE should be suitably diluted before use in such reactions. The DNA obtained was stored at −20°C until further use.

### Quality, quantity, and purity assessment of isolated DNA

Using all three of the above-mentioned different methods, the genomic DNA was extracted and was subjected to quality testing using a 1 kb DNA ladder (GeNieTM, Bangalore, India), and electrophoresis on a 0.8% agarose gel using 1× TBE buffer. The gel was stained with ethidium bromide (0.5 μg/ml) and visualized in the gel documentation system (GelDoc-It Imaging system, UVp model LMS-20E, Upland, USA). The yield of DNA (in terms of µg/ml) and purity were determined through a UV-visible spectrophotometer at 260 nm and 280 nm by calculating the absorbance ratio at A260/280 nm wavelengths.

### Efficacy towards PCR amplification through microsatellite markers

Despite the extracted DNA’s high quality assessed as above, the presence of PCR inhibitors might render further studies challenging by impeding the activity of Taq-polymerase in the PCR. Thus, PCR analysis of SSR markers was used to test the quality of the extracted DNA samples.

Touchdown PCR was performed using a thermal cycler machine (Eppendorf Mastercycler Nexus). Primer SRGMS310 was used (forward sequence: 5′-ATAACCCATGTCCTGCCCAA-3′ and reverse sequence: 5′-GAATGGCCATGATTTGCCCC-3′) [6]. The amplification was performed in a 15 µl PCR reaction mixture, containing 30 ng of template DNA, 7.5 µl of 2× Taq mix (Composition: 2× Taq buffer (Cat. # 786-447, 786-448, 786-449; G-Biosciences), 0.4 mM dNTPs, 3.2 mM MgCl_2_ and 0.02% bromophenol blue, 0.1–1 µg of both forward and reverse primers, and nuclease-free sterile water to make up the volume. The PCR conditions used, were as follows: initial denaturation at 94°C for 3 min, followed by 35 cycles of 94°C for 30 s, primer-specific T_m_ range for 30 s (i.e. 61.1°C), annealing at 72°C for 45 s; and a final extension at 72°C for 3 min. The PCR products were resolved by molecular weight in 3% high-resolution agarose to check polymorphism (Make: Sigma-Aldrich).

## Results and discussion

**Figure 1.**
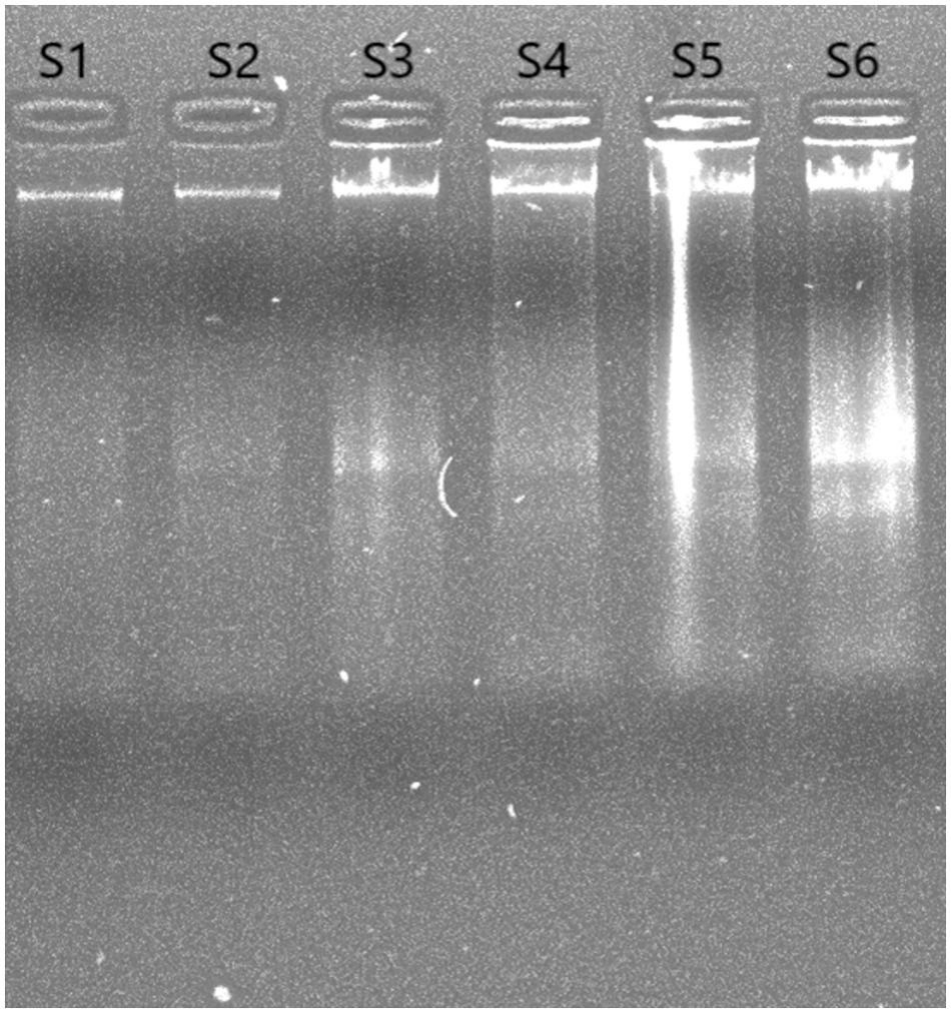
Agarose gel (0.8%) showing the isolated genomic DNA from leaf samples through all three discrete protocols. The samples labelled S1 and S2 employed the optimized double buffer protocol, S3 and S4 utilized the protocol by Sahu *et al.* [16], while S5 and S6 were processed using the CTAB method.

**Figure 2.**
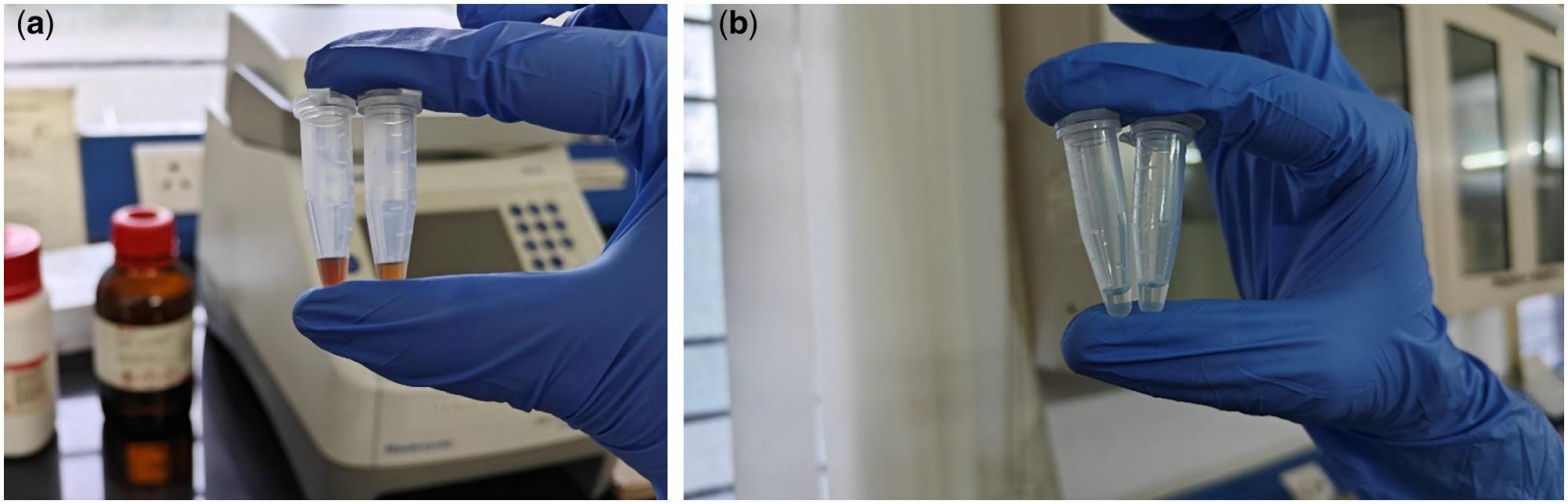
DNA observed after extraction through three distinct protocols: (a) brownish hue observed in the DNA extracted using protocols by Doyle and Doyle [15] and Sahu *et al.* [16]; (b) clear transparent high-quality DNA observed through our optimized protocol.

**Figure 3.**
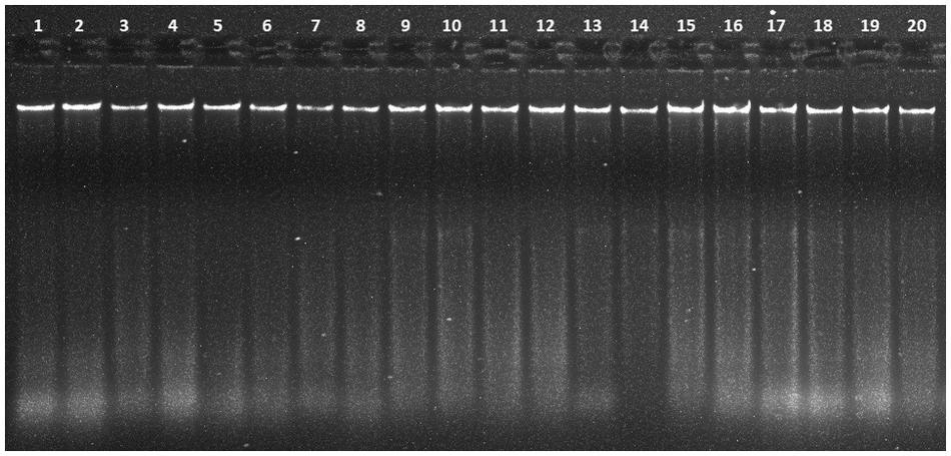
Extracted genomic DNA from 10 leaf samples (1–20) using the optimized double buffer method, separated by electrophoresis on 0.8% agarose gel.

The effectiveness of DNA extraction depends on both the tissue procurement and storage strategy [17]. Freshly collected leaves frozen in liquid nitrogen are preferred as they offer greater yield and purity of DNA. Even though CTAB is still one of the most widely used extraction techniques, it nevertheless has a somewhat low throughput. The DNA obtained through the Doyle and Doyle [15] and Sahu *et al*. [16] protocols was of poor quality, as smearing was observed (Fig. 1; samples S3–S6) and DNA was of brownish colour (Fig. 2a), which could be a consequence of *S. robusta* containing high level of polyphenols, which, when oxidized during extraction, are thought to covalently bind to DNA, giving it a brown hue and reducing its usefulness for PCR-based studies [8, 18]. As secondary products, polyphenols interfere with the genomic DNA isolation procedures and prevent further DNA amplification [19]. This resulted in the majority of the samples having poor DNA quality. In order to overcome this problem, a revised double buffer protocol was employed, ensuing improved DNA with higher quantity (ranging between 433 and 995 µg/ml), and clear transparency (Fig. 2b) when compared to the other two protocols (Table 2). The first [12] and second [15] protocols yielded A260/A280 ratios greater than 2 in most of the samples, which indicated RNA as an impurity (Table 2) as opposed to the protocol we developed [20]. This showed enhanced purity with A260/A280 values between 1.70 and 1.91, indicating the absence of polysaccharides, polyphenols, and RNA [21]. Further, the quality was found to be augmented when checked through gel electrophoresis where prominent and distinct bands were observed without any smearing (Fig. 3). This also illustrated how the double buffer method outperformed the other two procedures for the effective extraction and purification of DNA.

**Table 2.** The quantity and purity of leaf samples with all three protocols (P1: optimized double buffer protocol, P2: protocol by Sahu *et al*. [16], P3: CTAB method), were used to standardize the protocol 1 (optimized double buffer protocol) to isolate superior quality DNA from samples (S1–S6) of *S. robusta* species.

*Shorea robusta*		Fresh leaf samples	
Samples	Protocol	DNA purity (260/280)	DNA Yield (µg/ml)
S1	P1	483	1.79
S2	P1	446	1.82
S3	P2	301	1.49
S4	P2	296	2.12
S5	P3	218	2.05
S6	P3	303	2.22

**Figure 4.**
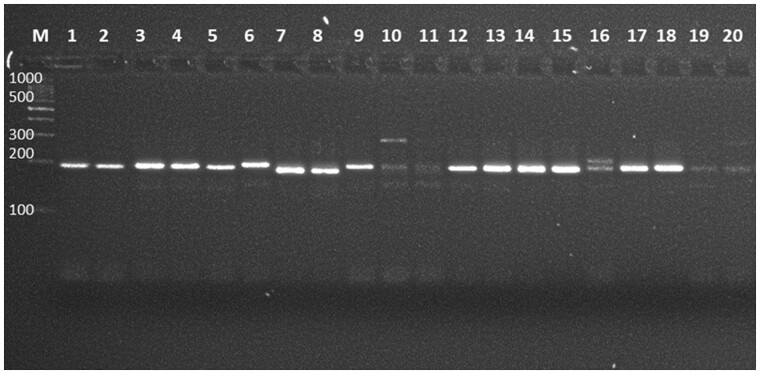
Representative sample (where M: 100 bp DNA ladder; 1–20 representing ten leaf samples) through SSRs showing polymorphic banding pattern in *S. robusta* using primer SRGMS310.

Accordingly, our revised extraction protocol was employed for subsequent analysis of all the leaf samples of *S. robusta* which generated high-molecular weight DNA with improved quality (Fig. 3) and purity (Table 3). An enhanced concentration of PVP was used during DNA extraction to enable effective PCR amplification and counteract the inhibitory effects of secondary metabolites, such as phenolic compounds, polysaccharides, and pigments. It inhibits DNA damage during the extraction process as it breaks the bond between DNA, RNA, and phenolics, and increasing DNA yield [22, 23]. Predominantly in species of forest trees, these secondary metabolites are known to hinder PCR amplification [24]. It has been reported that a high level of β-mercaptoethanol successfully removes the polyphenols, thus, an elevated concentration of β-mercaptoethanol was used [25]. Additionally, Weising *et al*. [26] stated that the shearing of DNA during extraction can directly or indirectly interfere with the enzymatic reactions. Thus, the absence of smears further substantiates the high purity of extracted DNA [26]. The quantity of NaCl and ascorbic acid was increased as compared to the other two protocols (Table 1) to remove the polysaccharides by increasing their solubility in ethanol [27]. PCR amplification employing SSR primers was carried out to assess the efficacy of the isolated genomic DNA using the aforesaid protocol (Fig. 4), which confirms its quality and purity. The primer pair employed, amplified the targeted region of the extracted DNA, and produced sharp and polymorphic bands, which verified the quality of the extracted samples. The approach proposed here may also be helpful for DNA extraction in other plant species with high levels of secondary-metabolic compounds and polysaccharides. Although a universal extraction protocol still remains unlikely, optimization experiments can improve our ability to achieve better extractions across plant groups of the family Diptercarpaceae, as we have demonstrated in the case of *S. robusta*.

**Table 3.** Quantitative estimates of extracted DNA from leaf samples of *S. robusta* using standardized DNA extraction protocol (P3: optimized double buffer protocol).

*Shorea robusta*		Fresh leaf samples	
Samples	Protocol	DNA purity (260/280)	DNA Yield (µg/ml)
1	P3	1.76	503
2	P3	1.70	646
3	P3	1.80	779
4	P3	1.81	806
5	P3	1.87	950
6	P3	1.78	902
7	P3	1.73	655
8	P3	1.82	584
9	P3	1.75	608
10	P3	1.79	433
11	P3	1.80	755
12	P3	1.75	742
13	P3	1.88	831
14	P3	1.91	816
15	P3	1.77	995
16	P3	1.80	701
17	P3	1.85	541
18	P3	1.72	689
19	P3	1.76	696
20	P3	1.74	811

## Conclusions

The optimized DNA extraction protocol reported here effectively yielded high quality and quantity of genomic DNA for *S. robusta* with very low levels of protein, RNA, polyphenol, and polysaccharide contaminants compared to other utilized protocols. The success of this reformed extraction method in obtaining high-quality genomic DNA from all the tested samples may have broader applicability such as for a wide array of molecular analysis, genetic engineering, phylogenetic analysis, and genome sequencing. All the advances standardized herein eliminate the need for repeated extraction, thereby reducing DNA extraction costs and assuring PCR fidelity. Hence, for high-throughput sample preparation suited for many DNA-based analytical methods, our protocol is broadly applicable, even in low-tech laboratory environments.

## Supplementary Material

bpad039_Supplementary_Data

## Data Availability

The data underlying this article are available in the article and in its online supplementary material.
