# Supplementary material for: An improved Shorea robusta genomic DNA extraction protocol with high PCR fidelity
Source: Biol Methods Protoc. 2023 Dec 9;8(1):bpad039. doi: 10.1093/biomethods/bpad039 (PMC10728041; doi:10.1093/biomethods/bpad039)
Supplement: bpad039_Supplementary_Data [file bpad039_supplementary_data.docx]

**Supplementary Table 1.** Geolocations of the sampled individuals.

| **Sl. no.** | **Samples** | **Latitude (˚)** | **Longitude (**˚**)** | **Altitude (m)** |
| --- | --- | --- | --- | --- |
| 1. | 1-2 | 29°32'57.1" | 79°11'58.8" | 979 |
| 2. | 3-4 | 29°32'30.7" | 80°05'43.1" | 873 |
| 3. | 5-6 | 29°29'77.2" | 80°06'53.2" | 801 |
| 4. | 7-8 | 29°07'40.6" | 80°05'17.8" | 1191 |
| 5. | 9-10 | 29°29'58.6'' | 80°06'32.0'' | 786 |
| 6. | 11-12 | 29°30'43.7" | 80°07'36.3" | 664 |
| 7. | 13-14 | 29°44'59.4'' | 80°20'53.3'' | 985 |
| 8. | 15-16 | 29°44'58.0'' | 80°21'28.3'' | 815 |
| 9. | 17-18 | 29°47'43.1'' | 80°08'19.1'' | 888 |
| 10. | 19-20 | 29°47'37.5'' | 80°08'37.9'' | 1007 |
